# Wave propagation in the marginal ice zone: connections and feedback mechanisms within the air–ice–ocean system

**DOI:** 10.1098/rsta.2021.0251

**Published:** 2022-10-31

**Authors:** Jim Thomson

**Affiliations:** Applied Physics Laboratory, University of Washington, Seattle, WA 98105, USA

**Keywords:** ocean waves, sea ice, air–sea–ice interaction

## Abstract

The propagation of ocean surface waves within the marginal ice zone (MIZ) is a defining phenomenon of this dynamic zone. Over decades of study, a variety of methods have been developed to observe and model wave propagation in the MIZ, with a common focus of determining the attenuation of waves with increasing distance into the MIZ. More recently, studies have begun to explore the consequences of wave attenuation and the coupled processes in the air–ice–ocean–land system. Understanding these coupled processes and effects is essential for accurate high-latitude forecasts. As waves attenuate, their momentum and energy are transferred to the sea ice and upper ocean. This may compact or expand the MIZ, depending on the conditions, while simultaneously modulating the wind work on the system. Wave attenuation is also a key process in coastal dynamics, where land–fast ice has historically protected both natural coasts and coastal infrastructure. With observed trends of increasing wave activity and retreating seasonal ice coverage, the propagation of waves within the MIZ is increasingly important to regional and global climate trends.

This article is part of the theme issue ‘Theory, modelling and observations of marginal ice zone dynamics: multidisciplinary perspectives and outlooks’.

## 1. Introduction

The marginal ice zone (MIZ) is defined by the coexistence of ocean waves and sea ice, or by the proximity of sea ice to open water. Functionally, these definitions are similar, because ocean waves typically propagate into sea ice from open water through a region that is distinct from the main pack ice. MIZs exist year-round at the edges of both Arctic and Antarctic sea ice, though the locations and extents are heavily modulated by seasonal cycles.

Decades of research on wave propagation in the MIZ have produced a large knowledge base, much of which is reviewed and advanced in this theme issue. The theoretical bases are wide ranging, as are the field data with which to evaluate the various theories [1]. Laboratory experiments complement these efforts with controlled conditions, including recent work comparing regular and irregular waves (and associated nonlinear effects) [2]. Wave forecasting within the MIZ is now done operationally and at the global scale using a variety of methods, depending on ice type and conditions, and it is not yet clear which, if any, methods can be made universal [3]. Some of the lingering uncertainty is related to the sparsity of field observations, but this is changing with the advent of inexpensive sensors that can be more easily deployed [4]. The forecasts of waves within the MIZ are now being used to improve predictions of ice evolution and floe size distributions [5]. Such two-way coupled wave–ice forecasting systems are essential to accurately representing the polar regions [6], especially as the extent of the Arctic seasonal MIZ expands with a warming climate [7].

**Figure 1.**
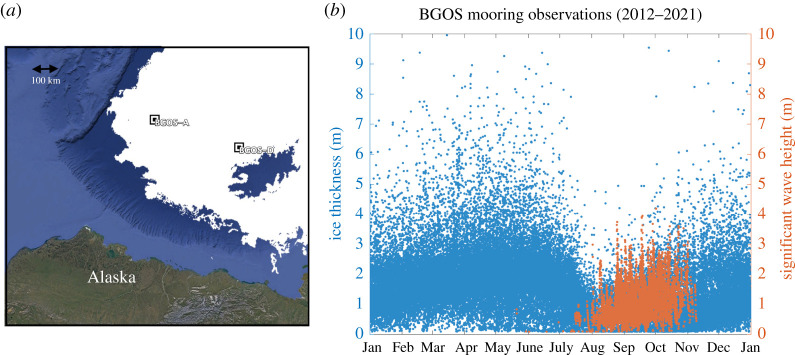
(*a*) Locations for BGOS moorings A and D, along with the ice extent on 1 October 2020. (*b*) Ice thickness (left axis, blue points) and significant wave heights (right axis, red points) measured hourly using moorings in the central Beaufort Sea from 2012 to 2021. (Online version in colour.)

At fixed observing locations, the seasonal evolution is a defining feature of the MIZ. Figure 1 shows an example, using nine years of observations from the central Beaufort Sea of the western Arctic Ocean. These data were collected from two of the moorings in the Beaufort Gyre Observing System (BGOS), as described in [8]. As the sea ice recedes in the early summer, waves form in open water. As the summer progresses, the waves increase—until the onset of refreezing in the autumn. Wave propagation in the MIZ occurs throughout each seasonal cycle, especially during the transitions (i.e. July and October). The ice extent shown in figure 1 is specific to 1 October 2020, but is representative of the autumn ice extent of the region. In other years, the BGOS mooring sites can be completely ice free at this time. Wave propagation is both a defining feature of the MIZ and a key process for the overall evolution of the air–ice–ocean–land system.

The intent of this review is to

(i) summarize present knowledge regarding wave propagation in the MIZ;
(ii) connect wave propagation in the MIZ to the larger air–ice–ocean–land system; and
(iii) describe key feedback mechanisms relating MIZ wave propagation to regional and global climate trends.

Wave and ice data from the BGOS moorings are used throughout to demonstrate the scales and magnitudes of these processes. This has an obvious bias of presenting signals most relevant to the western Arctic. Where possible, this is balanced by literature citations and information from the Antarctic and the eastern Arctic.

## 2. Wave propagation in the marginal ice zone

The canonical model for wave propagation in the MIZ is an exponential decay of spectral wave energy E(f) as a function of frequency f that reduces with distance x into the sea ice,

2.1 $$E(f,x)=E(f,0)\;e^{-\alpha (f)x},$$

in which α(f) is a spectral attenuation rate that depends primarily on ice coverage. This framework has been used extensively for decades [9,10], including incorporation into wave forecast models [11–13].

### (a) Attenuation rates

The attenuation rate α(f) is typically described as a power law that increases with frequency, $\alpha =af^{b}$ [14]. The practical result is that the shortest waves (i.e. the high-frequency wind–sea waves) are most rapidly attenuated, and thus farther within the MIZ only the longer waves (i.e. the swell) remain. This creates a general distinction based on the wave climates of the Arctic and the Antarctic. In the Arctic, fetches are short, wave energy is usually concentrated at f>0.1 Hz and waves are rarely observed more than 100 km into the MIZ [5,15]. In the Antarctic, fetches are long, wave energy is usually concentrated at f<0.1 Hz and waves are often observed well beyond 100 km into the MIZ [16–19].

**Figure 2.**
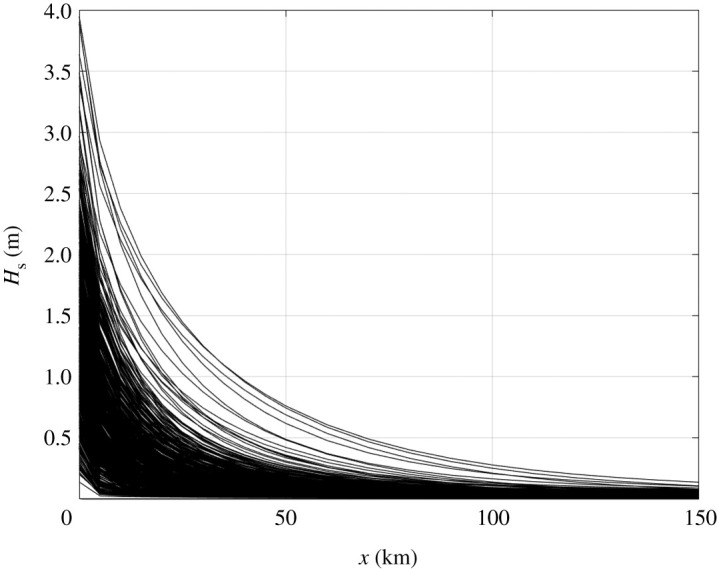
Theoretical attenuation of waves observed at BGOS moorings from 2012 to 2021. The significant wave height versus distance into the ice is calculated by applying a frequency-dependent attenuation rate to each observed energy spectrum E(f) and then integrating to determine the significant wave height at that location.

Figure 2 shows the theoretical attenuation of the waves measured at the BGOS moorings in the MIZ. Although only the bulk significant wave heights are shown, the calculation is spectral (equation 2.1), and the results use the conventional integration $H_{s}=4\sqrt{\int E(f)\;df}$. This theoretical attenuation uses $\alpha =0.3f^{2.7}$, which is consistent with prior observations from the region [15,20], and assumes that waves are normally incident with the ice edge (x=0) right at the mooring site. This overly simplified analysis is meant to simply show the typical scale of the problem; the Arctic MIZ is generally 5–50 km in extent, and waves are rarely energetic beyond 10 km. The Antarctic MIZ, by contrast, can be much larger in extent, with energetic waves beyond 100 km [18].

Obscured by the bulk $H_{s}$ results of figure 2 are the changes to the spectral shape within the MIZ. The preferential attenuation of energy at high frequencies (and thus short wavelengths) has a notable effect on the character of the waves. Without the short waves deep within the MIZ, long waves have a very smooth appearance in time and space and a narrow energy spectrum. This narrow spectrum causes the emergence of strong wave groups, with plausible mechanisms that are linear [21,22] and nonlinear [23]. The nonlinear dynamics are distinct from those in open water, because the wave steepness in sea ice is reduced and the dynamics are controlled by the propagation medium [24].

The lack of energy at high frequencies far within the MIZ has been a measurement challenge for decades, with long-running debates regarding the inference of a ‘roll-over’ in the spectral attenuation rate. Recent re-analysis of several published datasets indicates that most high-frequency measurements far within the MIZ are contaminated by poor signal-to-noise ratios, such that reported roll-overs are spurious [25]. Direct input to high frequencies by wind within the MIZ, which is discussed in a later section of this paper, remains another explanation for apparent roll-overs in attenuation.

Although less-well constrained than the frequency dependence, attenuation rates also have a strong dependence on ice type, ice coverage, ice thickness and floe size distribution. Ice concentration is the most common input to models [11], though ice thickness and floe size distribution have also been implemented [26]. Ice type is perhaps the most challenging dependence to address, since ice type is somewhat subjective. Visual classification of ice type has been successful in separating inferred attenuation rates [27], but such images are not always available. A related challenge is the heterogeneity of sea ice, which can be both difficult to quantify in field experiments and under-resolved in model grids. In particular, there is observational evidence suggesting that wave attenuation is strongest right at the edge of the MIZ (i.e. within a few wavelengths) relative to large-area averages [20]. This suggests some nonlinearity in the dynamics, or a specific set of processes at the ice edge.

### (b) Dissipation or scattering?

There is a long-standing debate over the physical mechanisms of wave attenuation in sea ice and their proper mathematical representation [28–30]. The attenuation itself is empirical: there is simply a net change in wave energy from $x_{1}$ to $x_{2}$. The mechanisms may be non-conservative (i.e. dissipation of wave energy) or conservative (i.e. scattering of wave energy). The mechanisms and associated mathematical frameworks are reviewed in this issue [1]. The representation in wave models is distinct: the non-conservative mechanisms alter the imaginary part of the complex wavenumber, while the conservative mechanisms alter the real part of the complex wavenumber. In such a framework, conservative processes can cause notable changes in wave direction (e.g. refraction) while non-conservative processes cause attenuation.

Recent observational work suggests that dissipation is the dominant mechanism, in which wave energy is lost by the breaking up of sea ice [31] and by turbulence at the ice–ocean interface [32]. Additional energy may be dissipated by the collision of ice floes [33]; the modelling of these mechanisms is reviewed in this issue [34]. Further evidence for negligible scattering (and thus strong dissipation) is provided by weak changes in the dispersion relation, relative to open water [35]. There is some evidence for non-negligible scattering via changes to directional distributions as waves propagate through the MIZ [36], but there are few observational studies that show strong evidence for scattering.

The distinction between dissipation and scattering mechanisms is important, even though either process can result in attenuation. When waves are dissipated, energy and momentum are released from the waves and cause dramatic changes to the ocean–ice system. In observations, this is difficult to discern from wave measurements alone, but measurements of ocean turbulence and ice motion can provide proxy evidence for energy and momentum transfers [37]. Fully coupled forecasts must account for these transfers of energy and momentum from the waves to the rest of the system. Moreover, fully coupled forecasts must also represent transfers of energy and momentum into the waves (i.e. wave generation within the MIZ).

### (c) Wave generation in partial ice coverage

In addition to processes that decrease (attenuate) wave energy in the MIZ, wind forcing may increase wave energy locally. This complicates interpretation of attenuation rates, which are typically estimated by comparing observations of E(f,x) at different positions x. Local wind-wave generation is likely as long as the MIZ has a non-zero fraction of open water [38]. At present, most wave models typically multiply the wind input source term by the fraction of open water [39]. Another approach is to define an ‘effective fetch’ as a function of open water (or ice concentration) and non-dimensionalize the wave generation as a function of wind speed [40,41].

**Figure 3.**
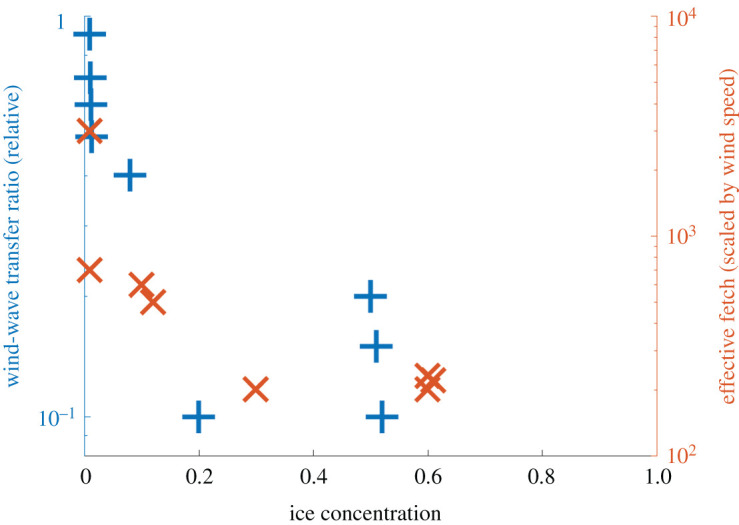
Non-dimensional parameters for wave generation as functions of partial ice concentration: wind work relative to open water (left axis, blue symbols) and fetch distance relative to open water (right axis, red symbols). (Online version in colour.)

The concept of effective fetch can be recast as an efficiency of wind work at the surface, relative to open-water efficiency [42]. Figure 3 combines the results of [40,42] as a function of ice concentration. Fundamental to this figure is the idea that waves provide the surface roughness that facilitates the wind doing work on the ocean. Thus both the local wind-wave generation and the local wind work on the ocean are reduced, but not zero, in the MIZ. A comprehensive treatment includes not only the ice concentration but also the relative magnitude of the wave orbital motions to the ice drift velocities [43]. Of course, the sea ice also alters the surface roughness that modulates wind work [44], but those effects are beyond the scope of this review.

Additional analysis of the BGOS observations in this theme issue demonstrates the importance of wind-wave generation in partial ice coverage and leads within the MIZ [5]. Noting that waves incident from open water rarely propagate farther than 100 km into the MIZ of the western Arctic (figure 2), this work finds that wave activity deep within the MIZ is almost entirely locally generated. These waves remain small ($H_{s}<0.5\;m$), because they are fetch-limited in leads and areas of partial ice coverage within the MIZ. Despite the small wave heights, these waves are still important to ice evolution and floe size distribution [5]. A key assumption in this work is that the local wind input to waves scales with the fraction of open water; this remains to be constrained with observations.

## 3. Waves in the air–ice–ocean system

A fundamental and universal treatment of wave propagation in the MIZ must include conservation of energy and momentum. The details for the wave physics depend on the relative importance of dissipation and scattering; the details for the rest of the air–ice–ocean–land system depend on what happens to that energy and momentum after being released from the waves. This section explores the processes that are driven by wave propagation into the MIZ. These processes are distinct from the local wind work discussed above; this section is concerned with the energy and momentum that waves carry to the MIZ from open water (i.e. a remote source, rather than a local source). A simple analogy is to think of the MIZ as a surf zone, in which the energy and momentum carried by waves are released to other processes.

**Figure 4.**
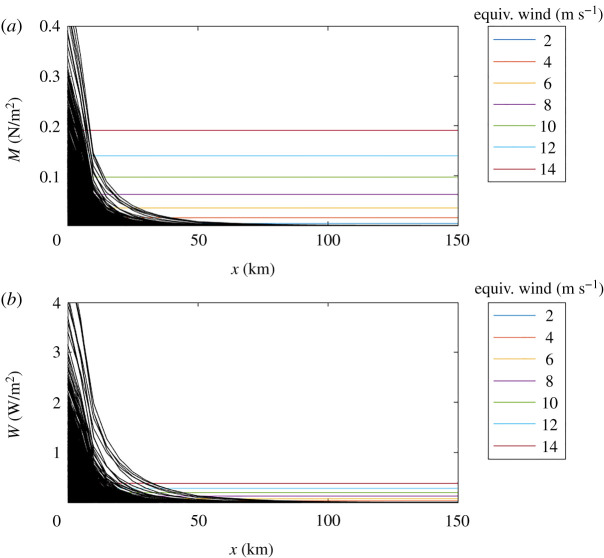
Rates at which wave momentum (*a*) and wave energy (*b*) would be released as functions of distance into the MIZ, as determined by the theoretical attenuation of waves observed at the BGOS moorings. Each panel includes the equivalent rates from direct wind forcing in open water at a range of wind speeds. (Online version in colour.)

Figure 4 shows the rates at which wave momentum and energy would be released according to the theoretical attenuation in figure 2 and also compares those rates to direct wind forcing in open water. The intent is to provide a dynamic definition of the MIZ as the region in which wave forcing exceeds local wind forcing and is thus a dominant signal. Details follow.

### (a) Wave momentum flux in the marginal ice zone

Waves carry a flux of momentum, usually referred to as the ‘radiation stress’, S, and gradients in this flux of momentum can drive mean circulation (and transport) in the MIZ [45–47]. The radiation stress is directly proportional to the wave energy, so that the rate of momentum transfer is

3.1 $$\dot{M}=\frac{\partial S_{ij}}{\partial x}∼\frac{1}{2}\frac{\partial \int E\;df}{\partial x}.$$

This is analogous to a stress, and figure 4 uses wind-stress estimates at several wind speeds to provide a relative comparison for the wave stresses in the MIZ. In the first 10 km of the MIZ, wave momentum regularly exceeds the momentum provided by strong winds. The momentum can be imparted to both the ice and the ocean, where it can drive transport and mixing that evolve the MIZ as a coupled air–ice–ocean system.

The magnitude and direction of this effective stress are dependent on the relative alignment of the wave propagation and the axis of the gradient, because the radiation stress is a tensor with four elements, ($S_{xx},S_{xy},S_{yy},S_{yx}$). For waves obliquely incident to the MIZ, the $S_{xy}$ can drive strong ($0.5\;m\;s^{-1}$) transport perpendicular to the gradient (and along the edge of the MIZ) [37]. In cases with waves at more normal incidence to the MIZ, the $S_{xx}$ can drive transport into the MIZ (and thereby compact ice floes at the edge of the MIZ) and create bands of higher concentrations of sea ice [48]. In either case, the momentum carried by the waves is released to cause significant changes in the transport and distribution of ice floes within the MIZ. The ice thickness has also been shown to be related to the wave radiation stress in the MIZ [49].

### (b) Wave energy flux in the MIZ

Waves carry a flux energy, and gradients in this energy flux are the rates at which energy is transferred from the waves to the ice and ocean of the MIZ. This can be described as a rate of work done,

3.2 $$\dot{W}=\frac{d\int Ec_{g}\;df}{dx},$$

where $c_{g}$ is the group velocity (which depends on frequency in the deep-water wave dispersion relation). Figure 4 shows the rate of work done by the waves through the theoretical MIZ of the BGOS data and compares it with the wind work of a range of wind speeds. The direct wind work is estimated as $c_{e}\tau$, where $c_{e}$ is an effective transfer velocity of around $2\;m\;s^{-1}$ [50]. The wave work can dominate for up to 50 km into the MIZ, relative to most moderate wind speeds. Thus, the wave work is significant over a larger extent of the MIZ than the wave momentum. This difference is explained by the group velocity $c_{g}$ contribution to the spectral energy flux (equation (3.2)); $c_{g}$ is faster at the low frequencies that propagate the farthest into the MIZ.

Recent works suggest that significant wave energy is lost in the form of turbulence in the upper ocean [32,37], though losses via ice floes fracturing [31] and ice floes colliding [33] may also be important. For the case where all wave energy is lost as turbulence, the depth profile of the turbulent dissipation rate ϵ(z) in the upper ocean will balance the gradients in the energy flux. For strong gradients, this remote source of turbulence may vastly exceed the local sources (i.e. wind) [42,43]. The wave-enhanced turbulence in the MIZ can increase the mixing of stratified melt-water, as well as the release of ocean heat during ice formation [51]. This turbulence is also significant for gas exchange in the MIZ [52]. There are also indirect changes to turbulence and mixing caused by waves. Langmuir turbulence, in which the sheared Stokes drift of the waves interacts with mixed-layer turbulence, is known to enhanced mixing [53]. The propagation of waves far into the MIZ thus provides another mechanism for mixing, which generally reaches deeper than the direct forcing (equation (3.2)) and can be particularly important when there is heat stored in the ocean [54].

## 4. Feedback mechanisms and climate trends

As the planet warms and sea ice losses accelerate, we must consider whether and how wave propagation in the MIZ impacts large-scale, long-term trends. Here, we examine how the processes described above manifest in the climate system and their associated impacts. We look for trends in how the sea ice affects the waves, how the waves affect the ice, and how these then impact the coasts.

**Figure 5.**
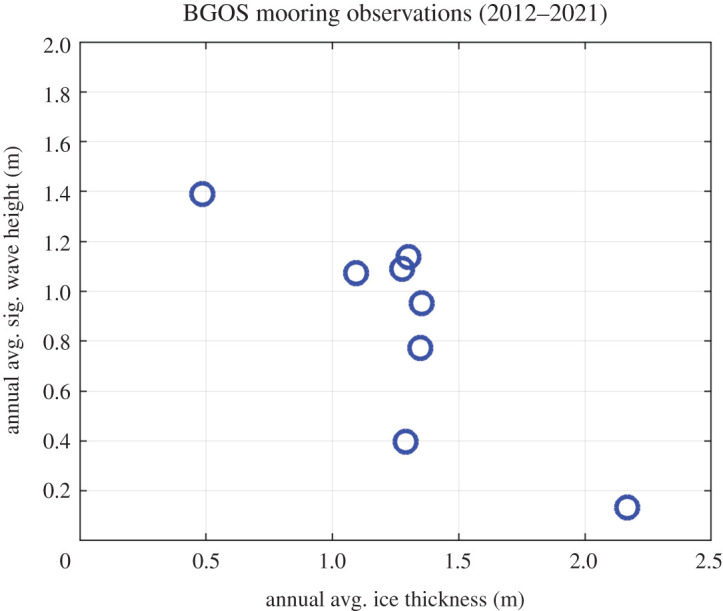
Annual average significant wave height versus annual average ice thickness at the BGOS moorings. (Online version in colour.)

Figure 5 uses annual averages from the BGOS dataset to show a relation between the waves and the ice at the mooring locations. The wave heights are greater, on average, when the ice is thinner, on average. This says nothing about the details of the physics, or even the dependence of attenuation rates on thickness. It simply shows that years with less ice have more waves.

### (a) Increasing wave climate

In the Arctic, it is now well documented that seasonal wave activity is increasing as a direct result of reduced sea ice and increased open-water fetch distances [55–60]. This trend includes not only the overall average but also increases in extreme wave events [61]. In the Antarctic, there are also linkages between the waves and the sea ice, specifically the break-up of sea ice by waves [16]. However, the fetches have always been large in the Southern Ocean [62], and thus a trend in wave activity is elusive.

The increasing wave activity means that more momentum and energy are transferred into an MIZ that is probably growing in extent [7]. Assuming canonical attenuation rates, larger waves will reach farther into the ice of the western Arctic, eventually beyond 100 km (figure 2). In addition to the associated changes in floe size distributions, ice transport and ocean turbulence, there are changes in ice formation. Pancake ice, which only forms in the presence of waves [63,64], has recently become a more common initial freeze up in the Beaufort Sea [65]. Whether this specific freezing process changes the overall seasonal cycle of ice thickness and strength in the region is as yet unknown.

### (b) Waves in ice loss events

Given that the increase in open-water fetch distances increases wave activity, there is hypothetical feedback mechanism in which waves cause ice retreat that continually increases the fetch (causing larger waves that cause further ice retreat). Although it is clear that storms can cause dramatic ice loss events [66,67], attributing a specific portion of the ice loss to wave forcing has been difficult. As figure 4 shows, the wind forcing is of a comparable magnitude, if not greater, at the large synoptic scales on which storms are modelled and analysed. Furthermore, winds and waves are usually co-temporal in a storm, such that isolating the forcing is difficult. More fundamentally, ice loss is often more driven by thermodynamics (i.e. heating and melting) than by mechanical dynamics (though those can enhance melting).

There are isolated examples of wave influences on ice loss, such as with Antarctic ice sheets [68] and in landfast ice [69]. More often, analysis suggests that combined atmospheric and oceanic stresses cause ice loss events [70]. In some cases, the ice loss from a given region is mostly a wind and wave transport process [71,72]. In other cases, waves break up sea ice and the ice is lost via enhanced melting [16]. Addressing the full range of processes requires wave–ice models fully coupled with air–ice–ocean system models [26,73].

### (c) Coastal impacts

In coastal regions, any waves not fully attenuated by sea ice may reach the shore. In this case, the analogy of the MIZ as a surf zone becomes explicit. The gradients in wave energy and momentum drive coastal processes, mostly notably near-shore circulation and sediment transport. Wave energy dissipated as turbulence in the surf zone may also increase water temperatures [74], which is particularly relevant for Arctic coasts [75].

Most Arctic coasts are eroding rapidly [76,77], and waves are a clear driver of these changes [78–81]. The presence and absence of sea ice controls the wave exposure at the coast, with strong regional variability [82]. Ice type is just as important as the presence/absence of ice, because this can control wave attenuation. In the spring, thick landfast ice causes complete attenuation and can prevent wave action at the coast for weeks or even months [83]. In the autumn, thin new ice causes only partial attenuation, and significant wave energy arrives at the coast [83]. This is consistent with other measurements of attenuation in landfast ice [84]. Thus, the specifics of the attenuation rates in different ice types are particularly important in coastal MIZs.

## 5. Conclusion

Attenuation of waves within the MIZ is a defining feature of the transition from open water to complete ice coverage. There is consensus that attenuation is a spectral process (i.e. per wavelength or frequency), and there are sufficient parametrizations of this process to be useful in wave forecasting. There is also an emerging consensus that wave energy and momentum are lost (i.e. dissipated) to the surrounding ice and ocean, rather than simply redirected (i.e. scattered). Much remains to be learned, including answers to the following questions.

— What is the dependence of the attenuation rate on ice type and thickness?
— How does heterogeneity in the ice affect wave attenuation?
— What nonlinear wave mechanics, if any, are important to attenuation?
— What controls the relative amount of wave energy lost to ice fracture/collisions versus to ocean turbulence?
— What balances the wave momentum lost to ice motion?
— How do local winds generate waves in partial ice coverage?

Answers to these process-level questions will support the continued development of fully coupled wave–ice–ocean–atmosphere models, which are needed for operational and climate forecasts in polar regions. The prior decades of work combining theory, experiments, observations and models provide productive examples to follow. The future holds more tools for observations, more realistic experiments and more computational power to incorporate this knowledge into the forecasts. Time to get back to work.

## Data Availability

The BGOS wave and ice data are available at http://hdl.handle.net/1773/46260.
